# The Baltimore Academy of Medicine

**Published:** 1887-01

**Authors:** 


### THE BALTIMORE ACADEMY OF MEDICINE.

Officially Reported for The Atlanta Medical and Surgical Journal.

Stated Meeting, November 16, 1886.

### INTRA-OCULAR CANCER IN A CHILD.

Dr. J. J. Chisolm had occasion to operate to-day on a case that was calculated to appeal to one's sympathies. The patient, a little girl of about two years of age, was brought to him by her mother in order that he might rectify, if possible, a peculiar expression that she had noticed in one of her child's eyes.

At a glance he noticed the yellowish appearance that is so characteristic of intra-ocular cancer. The mother stoutly refused to permit ophthalmoscopic examination until she was made aware of the serious nature of the trouble.

Upon examination he found nearly the whole of the eye filled with a gliomatous tumor formation, not, however, invading the optic nerve, but apparently confined to the retina and sub-retinal coats.

After consideration the mother consented to permit enucleation to be performed.

In the opinion of Dr. Chisolm there will, in view of the fact that the growth does not involve the optic nerve nor any of the socket tissues, not having broken through the outer coats of the ball, be no return; but should it have infiltrated the nerve or socket tissues the prognosis would have indeed been very grave.

The common history of these cases is that when they are present, there is a characteristic yellowish reflex that shows plainly through the pupil even without ophthalmoscopic aid. The growth continues to develop, the optic nerve becomes infiltrated, likewise the outer coats of the ball, until perforation and extension to socket tissues occurs. They are accompanied by great pain, increasing as the trouble progresses, and they not infrequently prove fatal in about one year unless aid is given sufficiently early. Recurrence is the rule after operation if the nerve or socket tissues are involved. In this case the doctor does not anticipate any return of the growth, as it was an unusually favorable case for operation. He has seen a child in which both eyes were affected by a similar pathological process. No operation was done, and when the child died its eyes were nearly the size of its head.

### FRACTURE OF CERVICAL VERTEBRÆ.

Dr. W. Chew Van Bibber said he had recently been invited by Dr. Robert Johnson to attend a post-mortem examination upon one of his patients who had died under rather peculiar circumstances.

The subject was a well-built man, a carpenter by occupation, who while pursuing his calling had a fall from which he recovered only partially, when he attempted to return to his work, but found it impossible to continue. He came to Baltimore for treatment and was under the care of Dr. Robert Johnson for four or five weeks previous to his death. There was a swelling on the back of his neck about the fourth cervical vertebræ, and besides this no other lesion could be found. He made no improvement upon treatment and finally died.

At the autopsy, made by Dr. Wm. T. Councilman, nothing was found to account for death until section of this swelling in the cervical region was made. Here there was found a complete solution of continuity of the bony column, the true nature of which was not then made out, as a histological examination of the tissues was necessary.

Dr. Van Bibber referred to several cases of fracture of cervical vertebræ that have occurred in his practice, but none of them behaved as this case did. They were all seriously affected from the first.

He referred to the case of Sir Charles Bell in which the patient, who had received an injury in the cervical region, in bowing himself from the surgeon's presence, completed the partly severed bony segments and dropped dead upon the floor.

### CALCIFICATION OF CORONARY ARTERIES.

Dr. James Carey Thomas said a case had recently occurred at the alms house that he considered interesting. The patient while walking across the floor suddenly dropped absolutely dead. Autopsy revealed calcification of coronary arteries and calcification elsewhere in the heart. It has been pointed out that in these cases, in which death is instantaneous, the trouble will usually be found in the heart.

Dr. J. J. Chisolm said deaths arising from lesions in the brain are never instantaneous.

Dr. Thomas said he was anxious to know what the autopsy had revealed as the cause of death in the case of Dr. Richardson, of Philadelphia.

Dr. Van Bibber related a very amusing experience that he had had in the early days of his medical career. He was suddenly called upon to do a post-mortem examination on a young colored women who had died suddenly on the street. He, after procuring the assistance of four of his young medical brethren, proceeded to find the reason of death. The autopsy was began at about 10 o'clock on a very warm summer morning and ended the next afternoon at 4 o'clock and he has not yet discovered what was the cause for the woman's death. His description of the method employed for removing the spinal cord was exceedingly amusing, especially, as after hours of labor the cord was removed an unrecognizable mass. He said that this experience had been recalled to his mind by the very strong contrast between the methods employed for these operations at that time and those used at the present date.

Dr. G. Lane Taneyhill said he did not think the improvements on these methods were all of very recent date, as in 1864, during his service in the civil war, the instruments employed at autopsies were of a very convenient design.

Dr. John Uhler has seen so much blundering by general practitioners, who pretend to make post-mortem examinations, that he has concluded that when an autopsy is to be made it should be referred to some one especially trained in this work.

Dr. James Carey Thomas said, as regards the case of fracture of cervical vertebræ referred to by Dr. Van Bibber, that he had been informed that no definite opinion as to the true nature of the bony lesion had as yet been given, and that the tissues were not undergoing manipulation preparatory to his histological examination.

### A CONTRIBUTION TO THE HISTORY OF HYDRAMNIOS.

Dr. Robert T. Wilson closed the exercises of the evening with a very exhaustive paper on the above title.

---

THE TRIALS OF A MEDICAL EXPERT.—Dr. F. A. Schmidt, writing in *Daniel's Texas Medical Journal*, relates his experience as an expert witness in a trial for rape. He and a brother practitioner were summoned about midnight by a deputy sheriff to examine the victim at her house, and the next day was commanded to go to the county-seat, ten miles distant, to repeat the examination. He was detained for a day or two, at his own expense, then allowed to go home, then summoned again and held for two days, and so on. The jury disagreed, and shortly after that the writer moved to another town 150 miles away. Here one day he was arrested and taken in custody of the sheriff to the town where the court was held. The trial was adjourned, but the witness was put under bonds of $500 to insure his next appearance. Now, in order to save his bond, he had to watch the case, telegraphing back and forth in order to keep himself advised of the doings of the court. Finally the case was dismissed, but not until Dr. Schmidt had spent over $200 in money, and lost more through the enforced neglect of his practice. He received no compensation whatever for his worry and trouble.—*Medical Record.*

